# An Integrated Inspection of the Somatic Mutations in a Lung Squamous Cell Carcinoma Using Next-Generation Sequencing

**DOI:** 10.1371/journal.pone.0078823

**Published:** 2013-11-11

**Authors:** Lucy F. Stead, Philip Egan, Aoife Devery, Caroline Conway, Catherine Daly, Stefano Berri, Henry Wood, Ornella Belvedere, Kostas Papagiannopoulos, Anderson Ryan, Pamela Rabbitts

**Affiliations:** 1 Leeds Institute of Cancer and Pathology, University of Leeds, Leeds, West Yorkshire, United Kingdom; 2 Gray Institute for Radiation Oncology and Biology, University of Oxford, Oxford, Oxfordshire, United Kingdom; 3 Department of Thoracic Surgery, St. James’s University Hospital, Leeds, West Yorkshire, United Kingdom; Virginia Commonwealth University, United States of America

## Abstract

Squamous cell carcinoma (SCC) of the lung kills over 350,000 people annually worldwide, and is the main lung cancer histotype with no targeted treatments. High-coverage whole-genome sequencing of the other main subtypes, small-cell and adenocarcinoma, gave insights into carcinogenic mechanisms and disease etiology. The genomic complexity within the lung SCC subtype, as revealed by The Cancer Genome Atlas, means this subtype is likely to benefit from a more integrated approach in which the transcriptional consequences of somatic mutations are simultaneously inspected. Here we present such an approach: the integrated analysis of deep sequencing data from both the whole genome and whole transcriptome (coding and non-coding) of LUDLU-1, a SCC lung cell line. Our results show that LUDLU-1 lacks the mutational signature that has been previously associated with tobacco exposure in other lung cancer subtypes, and suggests that DNA-repair efficiency is adversely affected; LUDLU-1 contains somatic mutations in *TP53* and *BRCA2*, allelic imbalance in the expression of two cancer-associated *BRCA1* germline polymorphisms and reduced transcription of a potentially endogenous *PARP2* inhibitor. Functional assays were performed and compared with a control lung cancer cell line. LUDLU-1 did not exhibit radiosensitisation or an increase in sensitivity to PARP inhibitors. However, LUDLU-1 did exhibit small but significant differences with respect to cisplatin sensitivity. Our research shows how integrated analyses of high-throughput data can generate hypotheses to be tested in the lab.

## Introduction

Lung cancer kills more people than colorectal, prostate and breast cancer combined [1]. Squamous cell carcinoma (SCC) constitutes 26% of all lung cancer [2], making it one of the main histological subtypes besides small-cell and adenocarcinoma. Karyotypes of lung SCCs have revealed some commonality in the genomic landscape of these tumours, including distal amplification of 3q [3] and a more focal amplification at 8p12 [4], but as yet these findings have not translated into the clinic. SCC remains the most common lung cancer histotype for which no genomically targeted therapy currently exists [5]. The lack of such therapy prompted inclusion of the lung SCC subtype in The Cancer Genome Atlas (TCGA) project, an international collaboration aimed at cataloguing cancer-driving genetic variation within tumours using multiple high-throughput approaches. One such approach was Next-Generation Sequencing (NGS), which has been used to gain insights into disease development and progression in several types of cancer, including both lung adenocarcinoma and small-cell lung cancer (SCLC) [6], [7]. The results of TCGA study of SCC revealed marked genomic complexity within lung SCC patient samples. However, pathway-specific alterations, hoped to yield therapeutic targets, did cluster by expression subtype, indicating the importance of integrating transcriptomic information in order to understand the phenotypic consequences of the plethora of genomic changes [8]. To understand how a more detailed, integrated analysis may aid inspection of lung SCC genomes, we deeply sequenced both the genome and transcriptome of LUDLU-1: a lung SCC cell line derived from a male patient whose smoking status is unknown. We also sequenced appropriate controls: the genome of an EBV-transformed lymphocyte cell from the same patient (cell line AGLCL) and the transcriptome of a normal bronchial epithelial cell line (LIMM-NBE1). To maximize our findings, we adopted an RNA sequencing method that captured both coding and non-coding RNA in a manner that retained information regarding the strand of origin. We have previously catalogued the transcriptional consequences of somatic structural variants in this cell line but here we focus on point mutations, aiming to see whether the mutational signature would give insight into disease etiology or carcinogenic mechanism, as it has for other cancer subtypes [6], [9], [10]. This type of in-depth characterisation of a given tumour can result in new hypotheses that can be tested using functional assays.

## Materials and Methods

### Cell Lines

LUDLU-1 and AGLCL were cultured as we have indicated previously [10]. LUDLU-1 was shown to be p63 positive and TTF-1 negative (data not shown) confirming a squamous carcinoma subtype. A549 was obtained from American Type Culture Collection (ATCC; Manassas, USA) and cultured in Advanced DMEM-F12 medium (Life Technologies, 1263-4010) supplemented with 5% foetal bovine serum (Sigma, F7524), 2 mM GlutaMAXTM (Life Technologies, 3505-0087) and 50 U/ml penicillin and 50 µg/ml streptomycin (Life Technologies, 15070) at 37°C with 7.5% CO_2_.

### DNA/RNA Extraction, Sequencing and Alignment

This was performed as previously described in Stead et al. [10]. Briefly, Complete Genomics used their proprietary method to sequence DNA that we extracted from the LUDLU-1 and AGLCL cell lines. RNA, extracted from LUDLU-1 and LIMM-NBE1, was sequenced by LGC Genomics on an Illumina HiSeq 2000 using 50 bp single end reads. Sequenced reads were aligned to the human reference genome, build 37, except in the case of miRNAs which were aligned to known miRNAs from build 36 using miRanalyzer [11]. All sequencing data have been submitted to the NCBI sequencing read archive (SRA: http://www.ncbi.nlm.nih.gov/sra) under accession numbers ERP001465 (LUDLU-1 and LIMM-NBE1 RNA sequencing) and ERP001771 (LUDLU-1 and AGLCL DNA sequencing).

### Mutation Detection

Complete Genomics call and score genomic variants during their local *de novo* assembly approach and output results into variant files, two of which (i.e. the tumour sample and matched normal) are compared, to identify somatic variants, using a tool called calldiff that is included in their proprietary cgatools 1.5.0, build 31, software suite. Somatic variants are each annotated with a somatic score [12]. Structural variants were extracted from the Junctions files, provided as part of the Complete Genomics sequencing report. Somatic mutations have been submitted to dbSNP (http://www.ncbi.nlm.nih.gov/SNP/) under the handle LIMM_YCR_PGG.

### Mutation Validation

50 coding and 50 non-coding single somatic variants were selected at random, and checked to ensure a normal distribution of somatic scores had been captured. Primer design failed for four but 96 underwent PCR and capillary sequencing, with seven giving ambiguous results. A receiver operating characteristic (ROC) curve was created using the validation results from the remaining 89 variants (data not shown), enabling us to set the somatic score threshold for single substitutions at 0.084, resulting in an estimated 100% specificity and 84% sensitivity. Indel validation was unsuccessful owing to ambiguous PCR/capilliary sequencing read-outs so all variants of this type were included if they had a somatic score >0. Somatic structural variants were validated as described in Stead et al. [10].

### Expression Analysis

Performed as described in Stead et al. [10]. Briefly, the number of reads aligning to exons, annotated as per Ensembl 60 [13], were counted and normalised by gene length and total number of mapped reads, resulting in a per gene expression metric: Reads Per Kilobase per million Mapped (RPKM). Reads that had between two and five valid alignments were assigned a single location using SEQEM [14].

### Single Somatic Mutation Analysis

A subset of 5% (108,362) of the germline polymorphisms in LUDLU-1 were selected, at random, for comparison with the somatic variants. All single nucleotide substitutions were annotated using a bespoke script that accessed Ensembl 60 via its Perl application programming interface. Additional information on the location of CpG islands was downloaded from the UCSC genome browser and incorporated into our in-house code. Pathogenicity scores were attained from MutPred [15]. Variable distributions or count data were tested between samples, or variant type, using an appropriate significance test (Wilcoxon and Chi-squared or Fisher’s Exact respectively) at the 5% level but corrected for multiple testing using the Bonferonni correction.

Allele counts for heterozygous variants were attained for both the genome and the transcriptome using SNVmix2 [16] (once Complete Genomics data had been converted using the cg2bam tools provided in the cgatools package). Allelic imbalance was tested only in those genes deemed as expressed in the tumour or normal and we required that a 10% change in absolute allele frequency been seen for either the DNA or RNA data, as per Tuch *et al*., 2010 [17]. Ratios were tested using Fisher’s exact test at a false discovery threshold of 5%.

To assess transcription-coupled repair, we used Cufflinks [18] to delineate transcripts within our LUDLU-1 RNAseq data, guided by Ensembl 60 gene annotations. The output included the strand each gene was located on. We recorded the number of mutations in each annotated, expressed gene, and whether each was on the transcribed or non-transcribed strand. We then calculated the rate of mutations per at risk base in the gene footprint. Significance (5% level with Bonferroni correction) and curve-fitting was performed using a zero-inflated, negative binomial regression model in R.

### Drugs

Cisplatin was purchased from Sigma (P4394), dissolved in dimethyl sulfoxide (DMSO) at 3.3 mM and stored at −20°C.

### Proliferation Assays

A flask of subconfluent cells was trypsinised; recovered cells were washed once with PBS and then seeded in culture medium at a density of 1,000 to 4,000 cells per mL in 96 well plates. The plates were incubated at 37°C with 7.5% CO_2_ overnight to allow cells to adhere. Treated culture medium was prepared at a 2X concentration and 100 µl was added to each well. The cells were then treated with one of six concentrations of cisplatin (0. 3125, 0.625, 1.25, 2.5, 5, 10 µM) or the control (DMSO). The plates were incubated for a further 5 days. At this point the cells were stained with crystal violet, allowed to dry and 100 µL of 33% glacial acetic acid was added. The absorbance was read at 590 nm using the POLARstar OMEGA plate reader. IC_50_ values were calculated using Calcusyn V2 software (Biosoft, Cambridge, UK). Cell line sensitivity to cisplatin was compared by ANOVA using SPSS Statistics v19 (IBM). Cisplatin was purchased from Sigma (P4394), prepared at 3.3 mM in DMSO and stored at −20°C.

### Clonogenic Survival Assay

The method was as described by Grenman *et al* [19]. In brief, a flask of subconfluent cells were trypsinised, washed once with PBS and seeded in culture media at a starting density of 1,200 cells per well in 96 well plates. A 1 in 10 serial dilution was made for A549 cells and for LUDLU-1, a 1 in 5 serial dilution was carried out. The plates were incubated at 37°C with 7.5% CO_2_ for 2 hours and then cells were exposed to 2, 4, 6 and 6 Grays of 137Cs (10 Gy, 1.958 Gy/min) in a GSR-D gamma irradiator. Plates were incubated for a further 7 to 14 days until colonies reached a size of 32 cells or more. Plating efficiency (PE) was calculated as described by Thilly et al [20] using Poisson statistics according to the formula PE =  -In (neg wells/total wells)/number of cells plated per well. The fraction survival was expressed relative to the PE of the un-irradiated control. Radiation survival curves were compared by linear regression using SPSS Statistics v19 (IBM) as previously described [21].

## Results

We sequenced the LUDLU-1 genome to an average coverage of 61x (i.e. each base was sequenced, an average, 61 times), and the matched lymphocyte to an average 55x. Copy number analysis shows that the SCC cell line is largely tetraploid and shares several features previously seen in lung SCC [10].

We identified 31,141 somatic single base substitutions (Supp. Table A in File S2), on average 10 per megabase (Mb). This exceeds the average mutation rate of 8.1 per Mb, ascertained from the sequencing of 178 lung SCC exomes as part of TCGA project in clinical samples [8]. A total of 181 somatic substitutions were located within coding regions of LUDLU-1 compared to an average of 360 in the series included in TCGA (Table 1). This included the only somatic mutation in the tumour cell line that is present in the Catalogue Of Somatic Mutations In Cancer (COSMIC) of clinical tumours [22]: a non-synonymous variant in *TP53* (ID:10656). This somatic mutation causes Arg248Trp; this is highly likely to inactivate the tumour-supressor function of p53, a protein involved in DNA repair, by removing the ability of Arg248 to directly contact the DNA response element via the minor groove [23]. No wild-type allele is present, as confirmed by the transcriptome sequencing. This specific p53 variant has been shown to be a gain-of-function mutation that promotes tumorigenesis [24].

**Table 1:** The consequence of LUDLU-1 somatic substitutions.

Consequence/Type	Variants	Transcripts affected	Genes affected
STOP_LOST	1	2	1
STOP_GAINED	3	4	3
UPSTREAM (5′+5 kb)	2100	3090	1749
DOWNSTREAM (3′+5 kb)	2202	3140	1822
SYNONYMOUS_CODING	52	149	44
NON_SYNONYMOUS_CODING	106	267	89
SPLICE_SITE 1–3 bps into an exon or 3–8 bps into an intron	19	48	21
ESSENTIAL_SPLICE_SITE First or last 2 bps of an intron	6	29	6
3′UTR	137	229	126
5′UTR	47	70	43
INTRONIC	11021	15822	4109
INTERGENIC	17642	NA	NA

We discovered 9431 somatic structural variants ranging from >1 bp substitutions to chromosomal translocations (Table 2 and Supp. Tables B and C in File S2). Included in the list of genes affected by structural variants is *BRCA2*, a tumour suppressor gene encoding a protein that repairs double-stranded (ds)DNA breaks by homologous recombination [25]. Therein a heterozygous single base deletion causes a frameshift anticipated to result in a dysfunctional protein product.

**Table 2:** Structural somatic variants identified in LUDLU-1.

Somatic Variant	Total	Within/affecting genes
Substitutions (>1 bp)	584	41
Insertions	4054	57
Deletions < = 100 bp	2622	32
Deletions >100 bp	66	42
Inversions	47	18
Duplications	29	18
Translocations	29	15

### LUDLU-1 Lacks the Mutational Signature Previously Associated with Tobacco Exposure

The smoking status of the LUDLU-1 donor is unknown. Previously, the smoking status of a small cell lung cancer cell line donor was assigned via the presence of a mutational signature of tobacco exposure [6]. This signature consists of an excess of G.T mutations, where G.T denotes a GC base pair being mutated to a TA base pair, and has been observed in both SCLC (34% prevalence) and lung adenocarcinoma (46% prevalence) whole genome sequencing data [6], [7], [26]–[29]. In contrast, the most common variants in LUDLU-1 were A.G (26%), and G.A transitions (24%). This implies that the LUDLU-1 somatic profile is not consistent with a smoking-based etiology for this lung SCC. However, to date, the smoking-associated somatic signature has not been validated on a genome-wide basis for lung SCC. We, therefore, downloaded data on 123,778 somatic mutations identified as part of TCGA study into clinical lung SCCs [8]. These somatic mutations originated from 163 current or previous smokers, and 7 lifelong never-smokers. Surprisingly, we found that G.T transversions were the most prevalent mutation subtype in both groups, with a significantly higher proportion in the never-smokers compared to the smokers (35% versus 33%: χ^2^, p = 2.4×10^−5^). We compared the distribution of G.T mutation proportions across the two groups and found no significant difference (Wilcoxon, p = 0.93). Tobacco carcinogens cause CpG.T transversions more frequently at methylated CpG dinucelotide [30] resulting in an expected increase in somatic CpG.T mutations outside of CpG islands, where 65% of CpGs are methylated compared to 15% within islands, in smokers. In contrast to this, the same proportion of somatic CpG.Ts were observed inside and outside of CpG islands in both the smoker and never-smoker groups from TCGA lung SCC data (Wilcoxon, p = 0.86). Hence, the mutational profile of tobacco exposure is a) lacking in LUDLU-1, and b) not seen exclusively in lung SCC tumours from smokers, according to TCGA data. We examine these results further in our discussion section.

### Sequencing the Transcriptome of a Lung SCC

We sequenced total RNA, after ribosomal RNA depletion, using a strand-specific method that enabled us to quantify both protein-coding and non-coding (nc)RNAs. To investigate tumour-specific expression patterns we first required a baseline of transcription in the appropriate non-diseased tissue, so we also sequenced a normal bronchial epithelial cell line that we established in-house and named LIMM-NBE1. A total of 600.4 million LUDLU-1 RNA reads aligned to the human reference genome; of these 87.5% (525.4 million) aligned uniquely, and an additional 168,546 small RNA (<20 bp) reads aligned to known miRNAs. Results were similar for LIMM-NBE1, where 700.8 million RNA reads aligned, of which 88.2% (618 million) did so uniquely, likewise a further 184,740 small RNA reads aligned to known miRNAs. To verify that the transcriptional profile of LIMM-NBE1 was characteristic of a bronchial epithelial cell, we inspected 27 genes listed on the Tissue Specific Gene expression Database (TiSGeD) as being highly bronchial epithelial cell-specific. There were sequenced RNA reads supporting each of these 27 genes in LIMM-NBE1; 24 (89%) exceeded the 10 RPKM threshold we used to denote expression and all but one of those exhibited above median expression in LIMM-NBE1 compared with all non-zero RPKM protein-coding genes [31]. This indicates that LIMM-NBE1 has a characteristic transcriptional profile for a bronchial epithelial cell. We performed the same analysis in LUDLU-1 and found that 23 of the 27 genes (85%) exceeded the RPKM threshold for expression and all but two of those exhibited above median expression in LUDLU-1 compared with all non-zero RPKM protein-coding genes. This indicates that LUDLU-1 did, indeed, originate in the lung.

### Additional Evidence of DNA Repair Deficiency

We used our RNA data to quantify expression in Reads Per Kilobase per million Mapped reads (RPKM), allowing us to inspect the relative abundance of each functional transcript class and to identify the transcripts, within each class, that exhibited the largest fold change in expression between the normal and the tumour (Supp. Table D in File S2). Interestingly the largest fold change was not for a protein-coding gene but an antisense gene called *RPPH1.1* (Ensembl ID ENSG00000259001) that appears not to be expressed in the tumour but exhibits an RPKM of 13,194 in the bronchial epithelium; this gene is antisense to *PARP2*. Expression of *PARP2* itself shows only modest differences between our samples (62 RPKM in LUDLU-1 and 98 in the normal) but antisense transcripts can regulate their mRNA counterparts post-transcriptionally, most often, though not exclusively, resulting in decreased protein levels [32], [33]. Consequently, reduced antisense transcription may lead to increased levels of protein product of *PARP2*, a DNA repair gene, in LUDLU-1.

We proceeded to integrate our datasets to investigate the incidence of allelic imbalance (AI), the unequal expression of two alleles at a heterozygous locus. AI is commonly observed in cancer as a result of genomic alterations such as copy number changes and loss of heterozygosity. We wished to investigate the situation when one allele is preferentially expressed owing to other mechanisms such as epigenetic changes affecting, or mutations within the regulatory regions of, one allele only. We were able to do this by identifying those heterozygous variants within our tumour cell line that had a significantly different allelic ratio in the RNA compared to the DNA sequencing data, acknowledging that this will indicate the presence, but not the cause, of the phenomenon. Of the 180,985 expressed heterozygous variants in LUDLU-1, 2.1% (3792) exhibit significant AI, affecting 1949 genes. Significantly more cancer genes [34], [35] contained one or more alleles that exhibit AI than would be expected by chance (χ^2^, p = 2.8×10^−5^), implying a role for this type of regulation in carcinogenesis. In total, 143 of the variants with AI are non-synonymous, with one being somatic and three being germline but located within cancer genes, as summarised in Table 3. We note that two non-synonymous, germline *BRCA1* variants in LUDLU-1 have imbalanced expression in favour of the mutant allele. In both cases the mutant allele is a genetic modifier of breast cancer risk [36]–[38], indicating that the resulting protein is altered in a manner that, whilst not able to cause disease in isolation, creates a predisposition. *BRCA2*, like *BRCA1* is a gene that has been causally linked to both breast and ovarian cancer. Both genes encode proteins that are involved in the repair of dsDNA breaks.

**Table 3:** LUDLU-1 non-synonymous variants that are either somatic (first row) or germline but in cancer-associated genes (last 3 rows).

Variant GenomicCoordinate	Gene	dbSNP ID	Basevariant	Amino acidvariant	DNA ref:varallelic ratio	RNA ref:varallelic ratio
Chr7∶99056827	*ATP5J2*	N/A (somatic)	T>C	T61A	32∶24	992∶19
Chr17∶41244000	*BRCA1*	rs799917	T>C	K1183R	18∶12	9∶33
Chr17∶41244936	*BRCA1*	rs16942	G>A	P871Q	31∶25	7∶29
Chr3∶158320703	*MLF1*	rs77911695	C>A	P201T	36∶10	2015∶155

Our findings highlighted several alterations in DNA-repair genes, leading us to further inspect the imprint of expression-linked repair in our data.

### Genome-wide, Transcription-coupled DNA Repair

**Figure 1:**
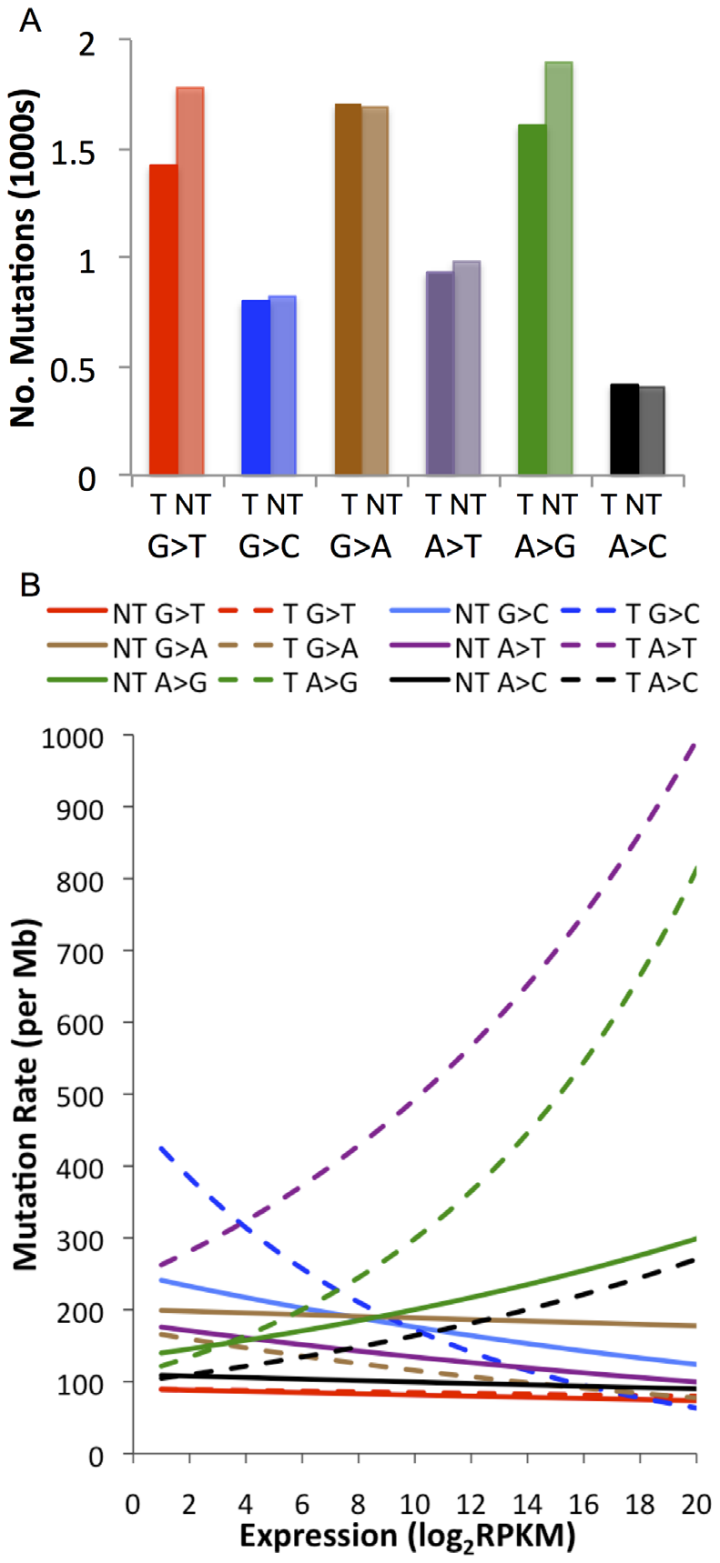
Expressed LUDLU-1 somatic mutations according to strand. a) The number of expressed mutations that appear on the transcribed strand (TS) or non-transcribed strand (NTS); b) The relationship between gene expression and mutation rate (mutations per Mb of at-risk bases in the gene footprint) for each mutation according to strand.

Transcription-coupled repair (TCR) in a SCLC was recently investigated by ascertaining the expression levels of genes harbouring somatic mutations using microarrays, and annotating expressed variants as being on the transcribed or non-transcribed strand, according to Ensembl gene annotations [6]. We wished to expand this analysis by looking at genome-wide expression, including that of novel transcripts assembled directly from our RNAseq data. Fig. 1A shows that, in accordance with previous findings, we observed more purine mutations on the non-transcribed than the transcribed strand; we found, however, the reverse to be true for G>A transitions. This indicates that TCR is in effect but, in the latter case, acting on the pyrimidine i.e. repair of C>T on the transcribed strand. We proceeded to investigate how gene mutation rates i.e. the number of mutations per at-risk base within a gene, alter with expression for each specific mutation. Here, our results different greatly from previous findings (Fig. 1B). We observed that mutation rates significantly change with gene expression for all types of variant, but that for A>T and A>C on the transcribed strand, and A>G regardless of strand. This relationship is positively correlated: an increased mutation rate is observed at higher expression levels. As the SCLC cell line analysis used Affymetrix U133A arrays, containing probes for 14,500 well-characterised human genes, 98% of which are protein-coding [6], we repeated our analysis using only protein-coding genes (Supp. Figure A in File S1). The resulting trends appear, overall, more similar to those that were previously seen but we did still observe an increase in mutation rate with increasing gene expression for C>T on the transcribed strand (i.e. G>A on the non-transcribed strand). These results imply that DNA repair mechanisms act differently between protein-coding and non-coding genes and suggest a potential reduction in the efficiency of TCR in LUDLU-1.

### Functional Assays

**Figure 2:**
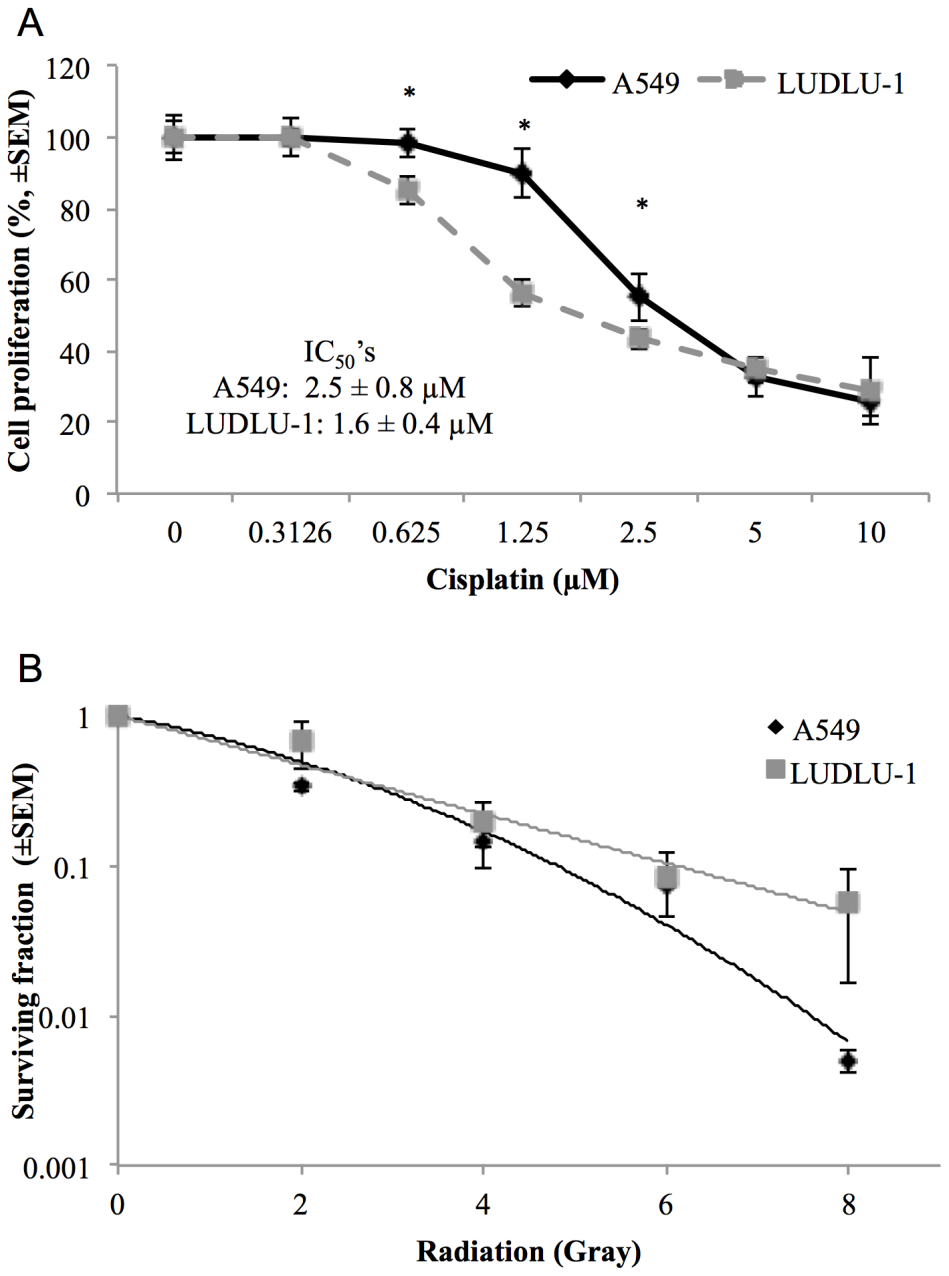
Molecular functionality testing of LUDLU-1. a) The effect of increasing dose of cisplatin on the cell proliferation of LUDLU-1 and A549; b) The survival fraction of LUDLU-1 and A549 when irradiated. Proliferation data is representative of duplicate independent experiments, with significance *p* values of less than 0.001. Radiation sensitivity data is representative of triplicate independent experiments.

To examine the DNA-repair functionality of LUDLU1 we determined the cell-line’s sensitivity to cisplatin and radiation relative to another non-small cell lung cancer (NSCLC) cell line, A549, which is diploid for the *BRCA1* locus and wild type for *TP53* [39]. In a 5-day proliferation assay LUDLU-1 has a 1.5 fold lower IC_50_ for cisplatin than A549 (1.6±0.4 and 2.5±0.8 µM, respectively) (Fig. 2A.). Whilst this is only a small change, the proliferation dose response was significantly different (p<0.01, ANOVA) between the cell lines. Pairwise comparisons of cell lines showed significant differences (p<0.001) between proliferation at doses of 0.625, 1.25 and 2.5 µM.

Clonogenic survival assays (Fig. 2B) showed a statistically significant difference in the radiation survival curves of the two cell lines (p = 0.011), although the difference was modest (90% cell killing at 4.8 Gy and 6.1 Gy for A549 and LUDLU-1, respectively) and was only clearly apparent at doses above 4 Gy. We also tested the effect of PARP inhibitors but found no alteration in sensitivity (IC_50_>10 µM, data not shown).

## Discussion

Tobacco smoke is the main risk factor associated with lung cancer, accounting for 70–75% of worldwide incidence [1]. However, incidence of lung cancer diagnoses in never-smokers is increasing in several countries [40], [41]. If classified as a separate sub-type, lung cancer in never-smokers would be ranked as the 7^th^ biggest cancer killer worldwide [42]. Research indicating that lung cancer in never-smokers constitutes a biologically distinct form of the disease has led to increased interest in this subpopulation as an understanding of its etiology may provide therapeutic targets, as is the case in adenocarcinoma [42]–[45]. We attempted to use a previously identified mutational signature of tobacco exposure [6], [26] to assign the smoking status of the lung SCC cell line originator patient. Whilst we found that LUDLU-1 lacked this signature, to our surprise we found that the somatic mutational profiles from never-smokers within TCGA data did not. This raises three possibilities. Firstly, the seven never-smokers in TCGA dataset have not been accurately annotated with regards their smoking status. Alternatively, the signature (i.e. G>T transversions as the predominating somatic mutation with altered proportions inside and outside of CpG islands) is not a consequence of exposure to tobacco carcinogens in lung cancers. Or, finally, there are biological mechanisms unique to this SCC line, and perhaps SCC clinically, that underlie the “signature” being associated with something other than tobacco exposure. The smoking status of patients from whom TCGA samples were acquired was self-reported; there is evidence of misclassification with such annotation [46], [47]. However, in order to ascertain how our findings affect the validity of a smoking-associated somatic mutational signature will require larger cohorts in which somatic mutations have been identified. We are, thus, unable to conclude the smoking status of the originator of the LUDLU-1 cell line, but cannot rule out an alternative etiology for this cancer. Whilst this manuscript was under review, a paper was published that describes signatures of mutational processes in cancer [48]. We compared the LUDLU-1 profile to those within that publication and found significant similarity with signature 5 (Supp. Figure B in File S1). Signature 5 was found in 93% of >150 lung squamous cell tumours tested, but often alongside signature 4, which significantly correlates with tobacco exposure. The etiology of signature 5 is unclear; there was some association with tobacco exposure but it was also prevalent in 7 other non smoking-associated cancers. The dominance of signature 5 in the LUDLU-1 somatic mutation profile (Supp. Figure B in File S1) suggests that it may be a good cell line model for testing the potential causes and consequences of this mutational profile of unclear origin.

Our approach was to perform an integrated analysis of the genome and transcriptome, as this can offer greater insight into pathogenic mechanisms than independent analysis of either dataset [49], [50]. Several findings implied that the LUDLU-1 genome has developed DNA-repair deficiency via a variety of mechanisms. A somatic substitution in LUDLU-1 is likely to lead to p53 inactivation. Knocking out *Trp53*, the ortholog in mice, does not directly cause cancer but increases the likelihood of spontaneous tumour formation [51] owing to defective DNA repair or apoptosis. A somatic deletion in LUDLU-1 is suspected to deactivate one copy of *BRCA2*. Being heterozygous, this deletion is unlikely to have caused the cancer in isolation, but could contribute to the formation of a defective DNA repair background. Similarly, allelic imbalance in favour of mutant *BRCA1* containing 2 germline variants associated with cancer risk could contribute to a DNA-repair deficient phenotype. Finally, we found reduced expression of a potential endogenous *PARP2* inhibitor. Genomic abnormalities resulting in loss of DNA repair function are associated with the development of several tumours, i.e. loss of *BRCA1* or *BRCA2* genes in hereditary ovarian or breast cancer [52]–[54], or defects in the DNA mismatch repair pathway in hereditary non-polyposis colorectal cancer [55]. In these tumours an alternative pathway(s), which may not be as effective, compensates the defect in an individual repair gene/pathway. This vulnerability makes these cancers more sensitive to therapies that inhibit DNA repair. To test if this was the case in LUDLU-1, we performed functional assays to assess cisplatin, PARP-inhibitor and radiation sensitivity, compared to a control lung cancer cell line. Whilst no changes were seen in the latter two, a modest but significant increase in cisplatin sensitivity was observed. Platinum-based chemotherapy regimes are a mainstay in the treatment of non-small cell lung cancer. However, treatment failure is often observed and is believed to be, in part, because of the upregulation of DNA repair pathways, which remove adducts caused by the platinum-based treatment [56], [57]. Tumour cells with BRCA loss have been reported to be ten to one hundred times more sensitive to cisplatin, PARP inhibitors and radiation [58]. The lack of radio-sensitivity and PARP insensitivity in LUDLU-1 cells suggest that the remaining wild-type copies of BRCA genes in this cell line provide sufficient functionality, or that alternative pathways can fully compensate for the heterozygous loss of functional *BRCA1* and increase in a single *BRCA2* allele, in response to these specific treatments. However, a deficiency in DNA-repair is apparent, and incompletely compensated to increase cisplatin-induced cell killing. This indicates that LUDLU-1 provides a good model for further experimentation into DNA repair phenotypes in the lung SCC subtype, the results of which can be evaluated in light of the DNA and RNA sequencing data provided.

The value of whole genome sequencing has been questioned when exon sequencing, possible with much larger sample numbers, can deliver tumour markers and drug targets [59]. However, our in-depth cross-platform analysis of a single sample has allowed us to speculate on underlying mechanisms of tumourigenesis and predict that hyper-mutation was caused by DNA-repair deficiencies.

## Supporting Information

File S1 **Supplemental Figure A. Expressed LUDLU-1 somatic mutations within protein-coding genes according to strand.** a) The number of expressed mutations that appear on the transcribed strand (TS) or non-transcribed strand (NTS); b) The relationship between protein- coding gene expression and mutation rate (mutations per Mb of at-risk bases in the gene footprint) for each mutation according to strand. **Supplemental Figure B. Mutational profiles for A) LUDLU_1, and B) Somatic Mutation Signature 5 as assigned in Alexandrov et al, Nature 2013 “Signatures of mutational processes in human cancer.”.** The 6 main substitution types are shown in different colours, broken down by sequence context i.e. one base either side of the mutated base. This shows how consistent the LUDLU-1 mutational profile is with signature 5. The lower image is reprinted by permission from Macmillan Publishers Ltd: Nature, copyright 2013.
(PDF)

File S2 **Supplemental Table A.** Somatic point mutations in LUDLU-1. **Supplemental Table B.** Somatic insertions (ins), deletions (del) and substitutions (sub) in LUDLU-1 that range from 2–99 bp. **Supplemental Table C.** Somatic structural variations in LUDLU-1including deletions >99 bp. **Supplemental Table D.** Expression levels in RPKM (Reads Per Kilobase per Million reads mapped) for the top 10 genes per functional transcript class that showed the largest increase or decrease between normal and tumour in terms of fold change.
(XLSX)
