# Supplementary material for: An Integrated Inspection of the Somatic Mutations in a Lung Squamous Cell Carcinoma Using Next-Generation Sequencing
Source: PLoS One. 2013 Nov 11;8(11):e78823. doi: 10.1371/journal.pone.0078823 (PMC3823931; doi:10.1371/journal.pone.0078823)

Supplemental Figure A. Expressed LUDLU-1 somatic mutations within protein-coding genes according to strand. a) The number of expressed mutations that appear on the transcribed strand (TS) or non-transcribed strand (NTS); b) The relationship between protein-coding gene expression and mutation rate (mutations per Mb of at-risk bases in the gene footprint) for each mutation according to strand.

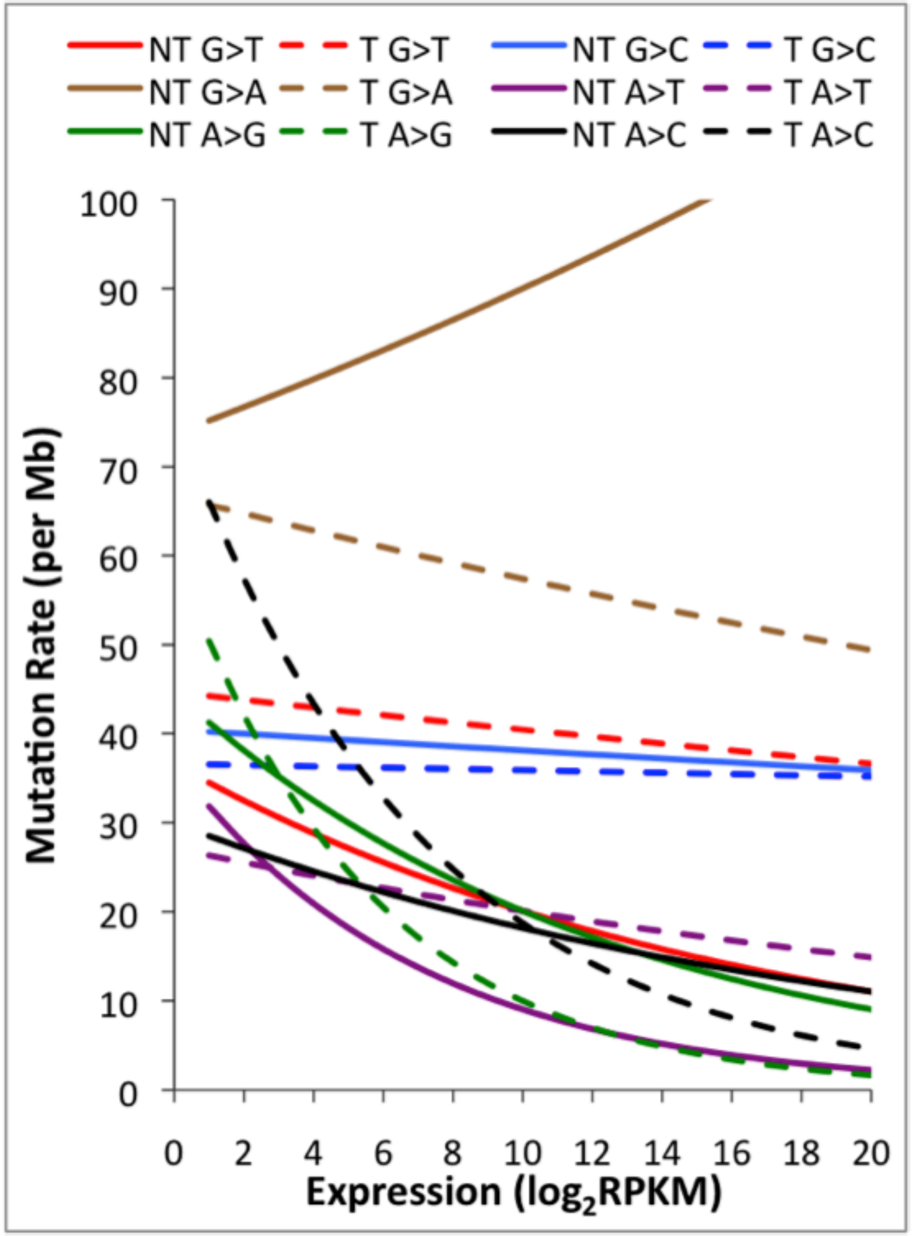

Supplemental Figure B. Mutational profiles for A) LUDLU_1, and B) Somatic Mutation Signature 5 as assigned in Alexandrov et al, Nature 2013 "Signatures of mutational processes in human cancer.". The 6 main substitution types are shown in different colours, broken down by sequence context i.e. one base either side of the mutated base. This shows how consistent the LUDLU-1 mutational profile is with signature 5. The lower image is reprinted by permission from Macmillan Publishers Ltd: Nature, copyright 2013.

A

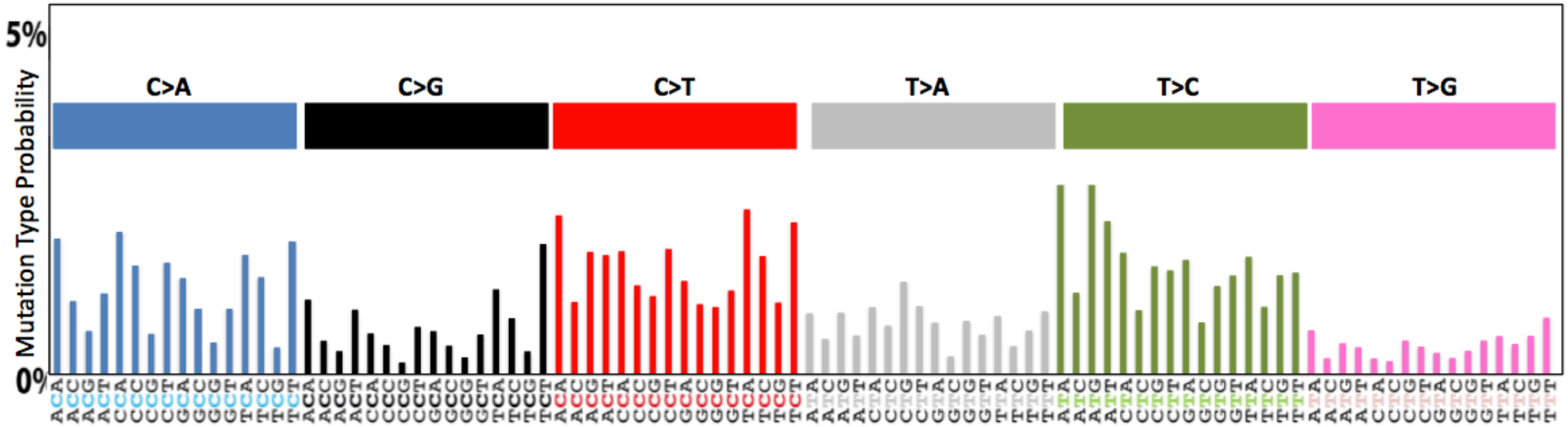

B

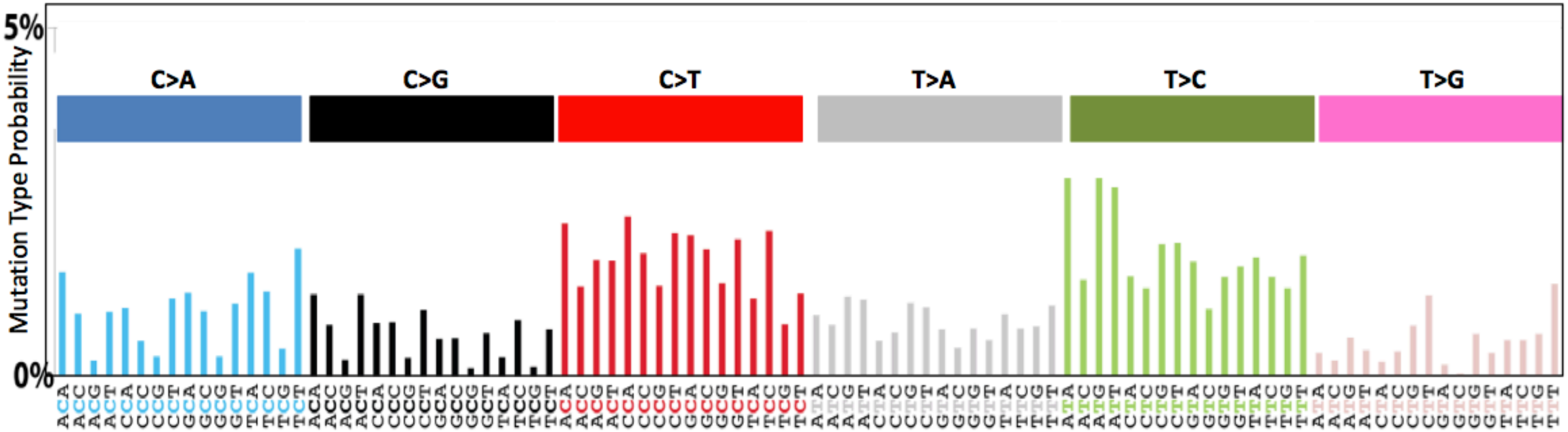

Supplement: File S1 — Supplemental Figure A. Expressed LUDLU-1 somatic mutations within protein-coding genes according to strand. a) The number of expressed mutations that appear on the transcribed strand (TS) or non-transcribed strand (NTS); b) The relationship between protein- coding gene expression and mutation rate (mutations per Mb of at-risk bases in the gene footprint) for each mutation according to strand. Supplemental Figure B. Mutational profiles for A) LUDLU_1, and B) Somatic Mutation Signature 5 as assigned in Alexandrov et al, Nature 2013 “Signatures of mutational processes in human cancer.”. The 6 main substitution types are shown in different colours, broken down by sequence context i.e. one base either side of the mutated base. This shows how consistent the LUDLU-1 mutational profile is with signature 5. The lower image is reprinted by permission from Macmillan Publishers Ltd: Nature, copyright 2013. (PDF) [file pone.0078823.s001.pdf]
